# Identification of a neutrophil-related gene expression signature that is enriched in adult systemic lupus erythematosus patients with active nephritis: Clinical/pathologic associations and etiologic mechanisms

**DOI:** 10.1371/journal.pone.0196117

**Published:** 2018-05-09

**Authors:** Joan E. Wither, Stephenie D. Prokopec, Babak Noamani, Nan-Hua Chang, Dennisse Bonilla, Zahi Touma, Carmen Avila-Casado, Heather N. Reich, James Scholey, Paul R. Fortin, Paul C. Boutros, Carolina Landolt-Marticorena

**Affiliations:** 1 Arthritis Centre of Excellence, Krembil Research Institute, University Health Network, Toronto, Ontario, Canada; 2 Division of Rheumatology, University Health Network, Toronto, Ontario, Canada; 3 Department of Medicine, University of Toronto, Toronto, Ontario, Canada; 4 Informatics and Bio-computing Program, Ontario Institute for Cancer Research, Toronto, Ontario, Canada; 5 Department of Pathology, Toronto General Hospital, University Health Network, University of Toronto, Toronto, Ontario, Canada; 6 Division of Nephrology, University Health Network, Toronto, Ontario, Canada; 7 Division of Rheumatology, Department of Medicine, Centre de recherche du CHU de Québec – Université Laval, Quebec City, Quebec, Canada; 8 Department of Pharmacology & Toxicology, University of Toronto, Toronto, Ontario, Canada; 9 Department of Medical Biophysics, University of Toronto, Toronto, Ontario, Canada; Peking University First Hospital, CHINA

## Abstract

Both a lack of biomarkers and relatively ineffective treatments constitute impediments to management of lupus nephritis (LN). Here we used gene expression microarrays to contrast the transcriptomic profiles of active SLE patients with and without LN to identify potential biomarkers for this condition. RNA isolated from whole peripheral blood of active SLE patients was used for transcriptomic profiling and the data analyzed by linear modeling, with corrections for multiple testing. Results were validated in a second cohort of SLE patients, using NanoString technology. The majority of genes demonstrating altered transcript abundance between patients with and without LN were neutrophil-related. Findings in the validation cohort confirmed this observation and showed that levels of RNA abundance in renal remission were similar to active patients without LN. In secondary analyses, RNA abundance correlated with disease activity, hematuria and proteinuria, but not renal biopsy changes. As abundance levels of the individual transcripts correlated strongly with each other, a composite neutrophil score was generated by summing all levels before examining additional correlations. There was a modest correlation between the neutrophil score and the blood neutrophil count, which was largely driven by the dose of glucocorticosteroids and not the proportion of low density and/or activated neutrophils. Analysis of longitudinal data revealed no correlation between baseline neutrophil score or changes over the first year of follow-up with subsequent renal flare or treatment outcomes, respectively. The findings argue that although the neutrophil score is associated with LN, its clinical utility as a biomarker may be limited.

## Introduction

Nephritis is a frequent disease manifestation in Systemic Lupus Erythematosus (SLE), affecting ~50–60% of patients. Lupus nephritis (LN) typically has a relapsing and remitting course, culminating in significant renal impairment in ~30% of patients and end-stage kidney disease in ~15% of patients [1–3]. This disease course poses significant difficulties for the treating clinician, who must balance the need to prevent renal damage with the complications of long term treatment with glucocorticosteroids (GCS) and immunosuppressives. Therefore, treatment is typically escalated when there is active inflammation in the kidney and tapered once this has resolved [4]. One of the impediments to this management approach is the lack of biomarkers forecasting development of LN or reflecting response to therapy [5]. This is further complicated by a significant subset of patients being relatively resistant to the current standard of care for LN.

Examination of RNA abundance profiles in the peripheral blood has proved to be an important means to identify potential biomarkers and novel pathogenic mechanisms in SLE, as evidenced by the discovery of the IFN gene signature using this technique [6, 7]. Recently, a study of blood transcriptional profiles in pediatric SLE patients found that elevated levels of neutrophil-related genes were associated with the presence of LN and global disease [8], and a similar association between these genes and LN has been observed in adults [9]. Here we used gene expression microarrays to directly contrast the transcriptomic profile of whole peripheral blood in adult active SLE patients with and without LN as a means to identify potential biomarkers and pathogenic mechanisms specific for LN. We confirm that the predominant transcripts that are overexpressed in active LN as compared to active non-LN are neutrophil-related, with the identified genes partially overlapping with those previously defined in pediatric SLE patients [10]. Using a second larger validation cohort, we confirm these findings and show that lupus patients with a previous history of LN have significantly lower levels of transcription of neutrophil-related genes, comparable to those seen in active non-LN. We further demonstrate that there is a modest correlation between the RNA levels of these neutrophil-related genes and the proportion of neutrophils in the peripheral blood, which is largely driven by the dose of GCS. Despite normalization of neutrophil-related RNA abundance in patients in renal remission, we find that changes in this gene expression signature do not generally parallel changes in clinical status over the first year following a renal flare, raising questions regarding the potential utility of this signature as a biomarker of renal inflammation.

## Materials and methods

### Ethics statement

The study was approved by the Research Ethics Board of the University Health Network (#05-0869-AE for subject recruitment and #05-0759-T for renal biopsy review), with all participants signing informed consent.

### Subjects and data collection

For the whole blood microarray studies, 38 patients satisfying 4 or more of the revised 1997 American College of Rheumatology classification criteria for SLE [11] were recruited from the University Health Network. Twenty-five had active LN, confirmed by renal biopsy at the time of the blood draw, with the remainder having active disease (score > 0 on the clinical (SLE Disease Activity Index-2000) SLEDAI-2K components [12]) and no clinical evidence of LN. ISN-RPS histopathological class [13] and activity and chronicity scores [14, 15] were determined by an individual renal pathologist (CA-C). Control blood samples (n = 17) were obtained from healthy donors with no family history of SLE, who were sex- (82% female) and age- (mean = 32.6 years; range 23–47) matched to the SLE patients. There were 192 participants in the validation phase of the study. Twenty-two were healthy controls (91% female; mean age = 35.4 years; range 23–58) and 170 were SLE patients: 89 active LN (61 with paired biopsies), 40 active non-LN, and 41 previous LN in remission. For the 10 patients who had repeated measures performed during follow-up after renal biopsy, clinical responses to treatment (complete response, partial response or treatment failure) were defined at 2 years following initiation of treatment, using previously defined criteria [16].

### RNA isolation, performance of microarrays, and validation of microarray findings

Total RNA was isolated from blood archived in PAXgene tubes utilizing the PAXgene Blood RNA Kit (Qiagen, Basel, Switzerland) with modifications to improve RNA yield and quality, as described previously [17]. Initial RNA profiling was performed using Affymetrix Human Gene 2.0ST arrays (Asurgen Services, Austin TX, USA). A total of 26 genes were selected for validation using 100 ng of RNA on the NanoString nCounter platform (Francombe Metagenomics Facility, McMaster University, Hamilton, Canada).

### Data processing and statistical analysis of microarray data

Raw CEL files were loaded into the R statistical environment (v3.2.5) and visualizations created using the lattice (v0.20–34) and latticeExtra (v0.6–28) packages. The data were processed using the RMA algorithm [18] using the affy package (v1.48.0) of the BioConductor library [19] and probes mapped using the EntrezGeneID map hugene20sthsentrezgcdf (v20.0.0) [20]. Arrays were evaluated for homogeneity using complete hierarchical clustering, as implemented in the cluster package (v2.0.4) with Pearson’s correlation being used as a similarity metric. A single array (AI_917_2703.CEL) was identified as an outlier in the normalized data, as seen in quality control plots, and was removed; remaining arrays were then re-normalized. A background intensity threshold was identified by evaluation of probes mapped to Y chromosome genes within female samples. Probes with intensity levels below this threshold (normalized intensity < 6) in all samples were removed (n = 8646). Of the remaining probes (n = 16087), those with a variance of normalized intensity values > 1 (n = 171) across samples were selected for visualization and the normalized data adjusted using row-wise mean centering with standard-deviation scaling. Scaled data were then subjected to DIANA agglomerative hierarchical clustering algorithm with Pearson’s correlation used as a similarity metric. Covariates were produced for SLE patient status: sex, type (renal/non-renal), disease status (SLE/control), renal biopsy class, and quantile-binning of patient scores (activity, chronicity). Filtered normalized intensities were correlated with available covariates (SLEDAI-2K scores, treatment status, renal biopsy class, activity and chronicity scores) across the cohort (Spearman's correlation followed by false-discovery rate (FDR) adjustment of p-values). To determine the effect of patient variables on transcript abundance, linear modeling was performed using the limma package (v3.28.21) for R with the following model:

$${\text{Y}}_{g}=\beta_{\text{SLE},\text{g}}{\text{X}}_{SLE}+\beta_{\text{REN},\text{g}}{\text{X}}_{REN}+\beta_{\text{Sex},\text{g}}{\text{X}}_{Sex}+\beta_{\text{Age},\text{g}}{\text{X}}_{Age}+\beta_{\text{DD},\text{g}}{\text{X}}_{DD}$$

where SLE indicates patient status (SLE or control), REN indicates renal status, and DD indicates disease duration. RNA abundance was compared between lupus and control subjects, as well as between renal and non-renal status, after allowing for differences due to sex, age and disease duration. Coefficients were fit for each effect and the standard errors of the coefficients were adjusted using an empirical Bayes moderation of the standard error [21]. To test if each coefficient was statistically different from zero, modified *t*-tests were applied, followed by FDR adjustment for multiple-testing [22].

### Data processing and statistical analysis of NanoString data

Raw data from NanoString, in the form of .RCC files, was loaded into the R statistical environment (v3.2.5) using functions provided in the NanoStringNorm package (v1.1.21) and normalized using the same package [23]. A total of 420 normalization methods were evaluated consisting of all possible combinations of methods available in NanoStringNorm. Outlier samples were identified at each step of the normalization process for all method combinations; if present, outlier samples were removed and the remaining data re-normalized. Methods were evaluated using a combination of sensitivity, specificity and dynamic range. Sensitivity was calculated as the proportion of endogenous probes identified as differentially abundant between disease and control samples (Student's *t*-test, p < 0.01). Similarly, specificity was calculated as the proportion of control probes (positive and negative controls provided by NanoString) identified as not differentially abundant between disease and control samples (Student's *t*-test; p < 0.01). Dynamic Range was calculated as the maximum median │log_2_ fold-change│ between disease and control samples across all probes. Methods were ranked using each evaluation metric and the rank product of all three methods used to identify the top performing normalization methods. The following normalization method was selected for use downstream: CodeCount (sum); Background (none); SampleContent (housekeeping.sum); OtherNorm (none). Housekeeping genes that were used for normalization included: *FPGS*, *GAPDH*, *HMBS*, *HPRT1*, *PPIB*, and *TBP*. Normalized data was subjected to unsupervised hierarchical clustering using divisive analysis (DIANA) with Pearson's correlation as a similarity metric to identify patterns. The resulting clusters were evaluated using the Adjusted Rand Index as available from the mclust package (v5.2) for R. Linear modeling was performed using the limma package (v3.28.21) with the following model:

$${\text{Y}}_{g}=\beta_{\text{ALN},\text{g}}{\text{X}}_{ALN}+\beta_{\text{ANLN},\text{g}}{\text{X}}_{ANLN}+\beta_{\text{RLN},\text{g}}{\text{X}}_{RLN}+\beta_{\text{CONTROL},\text{g}}{\text{X}}_{CONTROL}+\beta_{\text{Age},\text{g}}{\text{X}}_{Age}+\beta_{\text{Sex},\text{g}}{\text{X}}_{Sex}$$

where ALN (active lupus nephritis), ANLN (active lupus non nephritis), RLN (remission lupus nephritis) and Control are classification groups. Contrasts were applied to identify transcripts differentially abundant between sample groups. Standard errors of the coefficients were adjusted using an empirical Bayes moderation of the standard error [21] and model-based *t*-tests were applied to the coefficients, followed by FDR adjustment for multiple testing [22]. All visualizations were generated using the lattice (v0.20–34) and latticeExtra (v0.6–28) packages for R.

### Generation of a neutrophil score and statistical analysis of associations with clinical and laboratory parameters

To permit comparison with previous studies of neutrophil-related gene expression in SLE, the normalized log_2_ expression levels of the 9 neutrophil-related genes that overlapped with those previously published (*DEFA4*, *DEFA3/1*, *MMP8*, *CEACAM6*, *CEACAM8*, *LTF*, *MPO*, *ARG1*, and *MSHA3*) were summed to generate a neutrophil score. For continuous variables, the significance of association with the neutrophil score was determined by Spearman’s correlation coefficient. The significance of differences between two groups was determined using the Mann-Whitney *U* test and between more than 2 groups by a Kruskal-Wallis test followed by Dunn’s multiple comparisons test. Fisher’s exact test was used for comparison of proportions between groups.

### Characterization of neutrophil populations in the peripheral blood

For characterization of low density granulocytes (LDGs), peripheral blood mononuclear cells (PBMCs) were isolated from heparinized whole blood over a Ficoll gradient (GE Healthcare) and residual red blood cells lysed with hypotonic saline. The cells were then washed and stained with various fluorescence-conjugated antibodies including: anti-CD10-allophycocyanin (HI10a), -CD14- allophycocyanin/Cy7 (M5E2), -CD16-PE (B73.1), -CD15-BV605 (W6D3), -CD11b-PE/Cy7 (CBRM1/5), and -CD66b-Pacific Blue (G10F5), all from BioLegend. Approximately 150,000 lymphoid events were acquired per sample using an LSRII instrument (BD Biosciences), with dead cells being excluded by PI staining. The data were analyzed using Flow Jo software (TreeStar). LDGs were identified by their unique forward and side scatter profile together with expression of granulocyte markers (CD15^hi^CD10^hi^). Levels for positive staining were determined using fluorescence-minus-one (FMO) staining controls.

For some experiments the proportion of neutrophils within the whole peripheral blood cell pool was determined by staining with anti-CD10 and -CD15 following removal of red blood cells using hypotonic saline. The proportion of activated cells was determined by gating on CD11b^hi^CD66b^hi^ cells within the CD15^hi^CD10^hi^ neutrophil population.

## Results

### Interferon-induced genes predominate in the whole blood RNA abundance profile of active SLE patients but do not discriminate between the presence or absence of LN

Demographic, clinical and treatment information for the two cohorts of SLE patients are shown in Table 1. In the microarray cohort, there was no significant difference between active renal and non-renal patients in their demographics, mean SLEDAI-2K, or treatment, except that more of the renal patients were on GCS and the mean dose of prednisone was higher in this patient group.

**Table 1:** Demographics and clinical characteristics of study participants.

	Microarray		Validation		
	Active	Active	Active	Active	Inactive
	Non-renal	Renal	Non-renal	Renal	Renal
	N = 13	N = 25	N = 40	N = 89	N = 41
**Age: Mean ± SD**	29.4 ± 8.7	36.3 ± 10.9	31.9 ± 13.2	32.8 ± 12.0	37.4 ± 13.6
**Sex: No. Female (%)**	12 (92.3)	18 (72)	36 (90)	74 (83.1)	39 (95.1)
**Ethnicity: Caucasian (%)**	5 (38.5)	10 (40)	15 (37.5)	39 (43.8)	26 (63.4)
**Mean Disease Duration (years)**	5.9 ± 5.9	6.9 ± 8.1	7.8 ± 7.9	6.9 ± 7.0	14.6 ± 10.1
**Mean SLEDAI-2K**	10.9 ± 3.6	16.4 ± 8.6	9.3 ± 4.0	**14.4 ± 6.8**	1.8 ± 1.7
**CNS N (%)**	0 (0)	0 (0)	0 (0)	1 (1.1)	0 (0)
**Vasculitis**	4 (30.8)	3 (12)	12 (30)	6 (6.7)	0 (0)
**Arthritis**	8 (61.6)	3 (12)	22 (55)	16 (18.0)	0 (0)
**Myositis**	0 (0)	1 (4)	1 (2.5)	1 (1.1)	0 (0)
**Nephritis**	0 (0)	25 (100)	0 (0)	85 (96)^a^	2 (4.9)^b^
**Rash**	5 (38.5)	4 (16)	11 (27.5)	19 (21.3)	0 (0)
**Alopecia**	4 (30.8)	3 (12)	10 (25)	7 (7.9)	0 (0)
**Ulcers**	5 (38.5)	5 (20)	9 (22.5)	9(10.1)	0 (0)
**Pleuritis**	3 (23.1)	3 (12)	5 (12.5)	8 (9.0)	0 (0)
**Pericarditis**	1 (7.7)	3 (12)	2 (5)	7 (7.9)	0 (0)
**Low complement**	9 (69.2)	19 (76)	24 (60)	66 (74.2)	14 (34.1)
**dsDNA Abs**	11 (84.6)	20 (80)	26 (65)	66 (74.2)	18 (43.9)
**Fever**	0 (0)	0 (0)	3 (7.5)	3 (3.4)	0 (0)
**Thrombocytopenia**	0 (0)	1 (4)	3 (7.5)	5 (5.6)	0 (0)
**Leukopenia**	2 (15.4)	0 (0)	6 (15)	4 (4.5)	0 (0)
**Prednisone: N (%)**	6 (46.2)	**21 (84)**	18 (45)	**72 (80.9)**^c^	26 (63.4)
**Mean Dose Prednisone**	13.5 ± 16.5 (0–50)	**34.8 ± 26.2 (0–60)**	7.3 ± 12.5 (0–50)	**44.4 ± 94.1 (0–625)**	6.3 ± 7.1 (0–25)
**Anti-malarials: N (%)**	9 (69.2)	15 (60)	26 (65)	53 (59.6)	33 (80.5)
**Immunosuppressives: N(%)**	4 (30.8)	10 (40)	14 (35)	48 (53.9)	27 (65.9)
** Azathioprine**	1 (7.7)	3 (12)	3 (7.5)	13 (14.6)	14 (34.1)
** Mycophenolate**	1 (7.7)	7 (28)	5 (12.5)	**30 (33.7)**	13 (31.8)
** Methotrexate**	2 (15.4)	0	6 (15)	**1 (1.1)**	0
** Cyclosporine**	0 (0)	0	0 (0)	5 (5.6)	1
** Cyclophosphamide**	0 (0)	0	0 (0)	2 (2.2)	0

Ten of the active LN and 22 of the active non-LN overlapped between the microarray and NanoString cohorts. Significant differences for patient demographics, mean SLEDAI-2K, and treatment, between active renal and non-renal patients for each cohort are highlighted in bold.

^a^ of the 4 patients with negative renal findings at the time of biopsy, 2 had hematuria, 1 proteinuria, and 1 proteinuria + hematuria prior to the biopsy, as defined by the SLEDAI-2K.

^b^ there were 2 patients with stable proteinuria not attributed to active LN.

^c^ differences in prednisone use between active non-LN and active LN patients were due to the requirement to time the blood draw within ± 2 wks of the renal biopsy for active LN. Most active non-LN patients were recruited at the time of clinic visit and changes in treatment were initiated at the same visit. In contrast, the majority of active LN patients were treated prior to blood draw/renal biopsy (range: 1 day—~ 2 months). 15/17 active LN patients off prednisone had the drug initiated immediately following blood draw/biopsy (the remaining 2 had class V nephritis).

**Fig 1:**
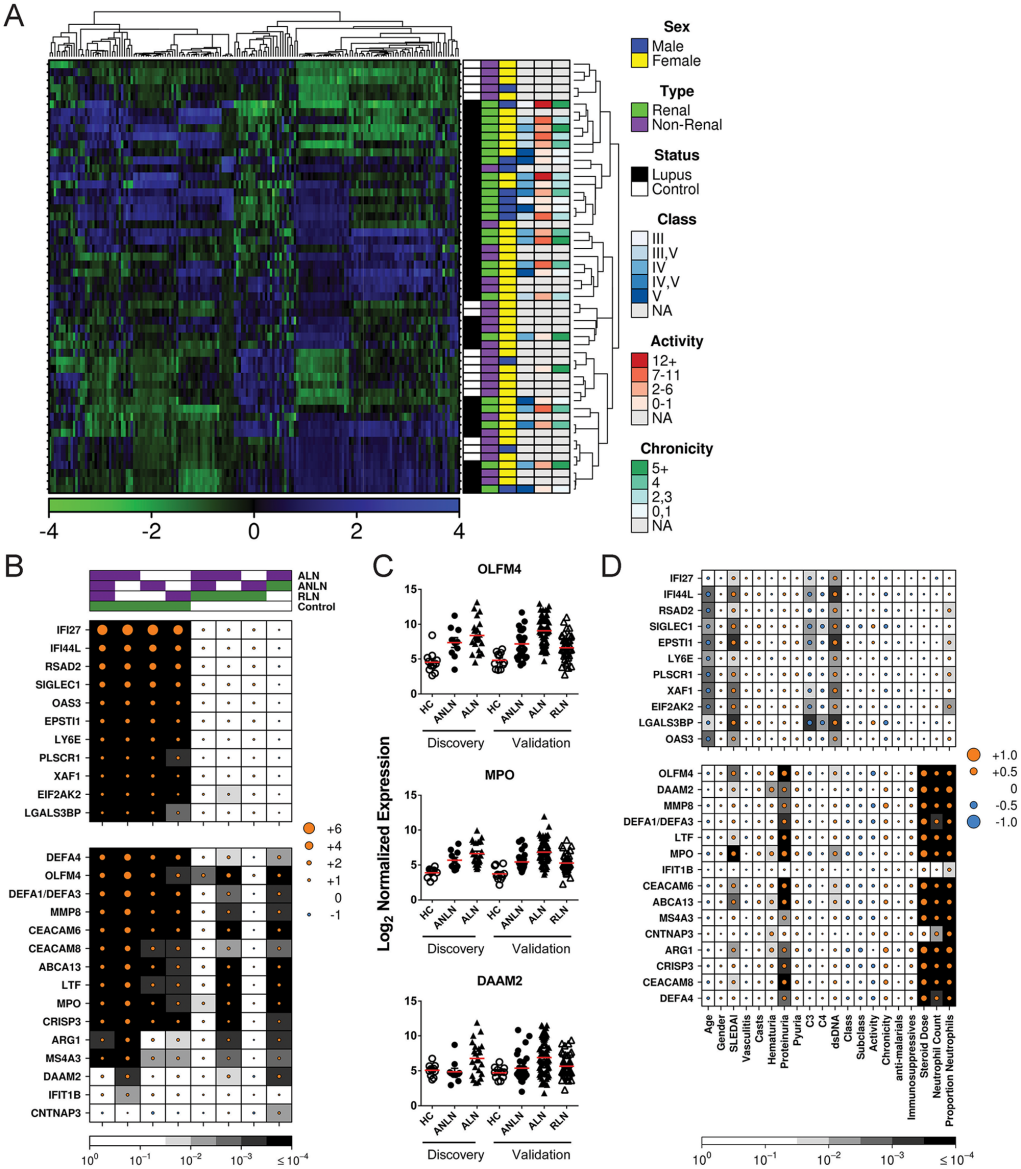
Differences in RNA abundance between patient groups. **(A)** Transcriptomic profiles for the 171 genes with the highest abundance variance, as determined by microarray, in the whole blood of 38 SLE patients and 17 controls are shown. Normalized signal intensities were adjusted using row-wise mean centering with SD scaling, with blue indicating over-expression and green indicating under-expression. Hierarchical clustering was performed on the samples (row) and genes (columns) using divisive analysis. **(B)** Log_2_ fold-change of normalized RNA abundance for comparisons between patient groups (ALN, active lupus nephritis; ANLN, active no lupus nephritis; RLN, remission lupus nephritis), as determined by NanoString. Top covariates indicate the experimental (purple) and control (green) groups being compared in each column. The size of the circles indicates the magnitude of the fold-change for each comparison, with orange indicating over-expression and blue indicating under-expression in the experimental group. The background in each cell indicates the statistical significance of the comparison as determined by multivariate linear modeling followed by FDR correction. Results for 11 IFN-induced genes are shown at the top of the figure and are separated from 15 genes examined that were identified as differentially expressed between renal and non-renal disease in the microarray study, which are shown at the bottom of the figure. **(C)** Results for representative neutrophil-expressed genes, showing the similarity between gene expression profiles in the discovery and validation cohorts. **(D)** Correlation of normalized RNA abundance with clinical and laboratory variables. Data is expressed similarly to that shown in panel B, except that the clinical variables assessed are shown at the bottom of the figure. Statistical significance was determined by Spearman correlation followed by FDR correction of p-values.

Hierarchical clustering of the 171 genes with the highest variance across the 55 subjects whose peripheral blood gene expression was examined by microarray revealed several distinct groups among the samples (Fig 1A). Covariates for which the clustering algorithm was best able to differentiate groups, as determined by the Adjusted Rand Index (a measure of clustering, with 0 indicating no agreement and 1 complete agreement), were renal disease (0.258) and sex (0.265). There was negligible clustering of SLE patients relative to controls. In addition, no clustering was observed based on renal biopsy class, activity score, or chronicity score. RNA abundance of individual gene transcripts was then evaluated between SLE patients and healthy controls and between SLE patients with and without LN, using linear modeling incorporating age, sex, and disease duration. Seventy-one genes were found to be differentially expressed between SLE patients and healthy controls (FDR < 0.1). Of the 33 genes that had increased RNA abundance (> 1 log_2_ fold-change) in SLE patients, all were IFN-induced (S1 Table). Thus, the predominant RNA abundance signature in the whole peripheral blood of SLE patients is similar to that observed for purified PBMCs in previous studies [6, 7].

### The gene expression signature associated with active LN is enriched for genes highly expressed in neutrophils

There were no genes that were differentially expressed between active SLE patients with and without LN at a threshold of FDR < 0.1; however, given the small number of samples examined it was deemed appropriate to examine less stringent thresholds. Therefore, we examined those genes that demonstrated a > 1 │log_2_ fold-change│ with a FDR < 0.25 (n = 27; Table 2). Of these, 22 were overexpressed in LN patients, and of the 18 genes whose expression pattern is known, all are expressed in neutrophils. Furthermore, 15 of these genes have been previously reported to be enriched in a specific subset of neutrophils called low density granulocytes (LDGs) that is found at higher levels in SLE patients [7, 24], 9 of which overlapped with those identified in the neutrophil module (M5.15) that was reported to be associated with LN in pediatric and adult SLE [8, 9].

**Table 2:** Genes differentially expressed between active SLE patients with and without LN.

					Renal vs Non-renal		Lupus vs Control	
Gene Symbol	Gene Name	Cell population/ function	Fold increase LDG^a^ [25]	M5.15 Module^b^	Fold- Change (Log_2_)	q value	Fold-Change (Log_2_)	q value
**OLFM4**	olfactomedin 4	LDG	29.41		1.80	0.160	1.19	0.717
**CEACAM6**	carcinoembryonic antigen-related cell adhesion molecule 6	LDG	17.4	Y	1.72	0.160	1.04	0.722
**CEACAM8**	carcinoembryonic antigen-related cell adhesion molecule 8	LDG	18.27	Y	1.63	0.172	1.22	0.692
**MMP8**	matrix metallopeptidase 8	LDG	41.5	Y	1.60	0.184	1.70	0.417
**LTF**	lactotransferrin	LDG	12.01	Y	1.58	0.172	1.04	0.773
**DEFA4**	defensin, alpha 4	LDG	25.57	Y	1.46	0.160	0.97	0.661
**DEFA3/1**	defensin, alpha 3/1	Neutrophil		Y	1.43	0.238	1.79	0.475
**DAAM2**	dishevelled associated activator of morphogenesis 2	Neutrophil/ Adhesion			1.43	0.160	0.04	0.997
**CNTNAP3**	contactin associated protein-like 3	Adhesion			1.32	0.155	-1.18	0.188
**MS4A3**	membrane-spanning 4-domains, subfamily A, member 3	LDG	17.85	Y	1.27	0.165	0.96	0.657
**ARG1**	arginase 1	LDG	2.35	Y	1.25	0.170	0.19	0.989
**MPO**	myeloperoxidase	LDG	14.66	Y	1.23	0.160	0.57	0.826
**ABCA13**	ATP-binding cassette, subfamily A, member 13	LDG	4.54		1.19	0.160	0.84	0.621
**CA1**	carbonic anhydrase 1	RBC/ Neutrophil			1.13	0.181	0.12	0.996
**IFIT1B**	interferon-induced protein with tetratricopeptide repeats 1B	IFN-induced			1.12	0.178	0.11	0.996
**CRISP3**	cysteine-rich secretory protein 3	LDG	12.18		1.12	0.170	0.61	0.835
**LCN2**	lipocalin 2	LDG	19.02		1.10	0.181	0.80	0.724
**BPI**	bactericidal/permeability increasing protein	LDG	16.59	Y	1.09	0.191	0.61	0.871
**LOC101927153**					1.08	0.160	-0.41	0.913
**XK**	X-linked K gene, Kell blood group	LDG	8.56		1.08	0.167	-0.02	0.999
**CNTNAP3B**	contactin associated protein-like 3B	Adhesion			1.05	0.155	-0.92	0.229
**ARHGEF12**	Rho guanine nucleotide exchange factor 12	LDG	2.81		1.00	0.160	0.02	0.999

^a^ as reported in cited reference. All fold increases are for LDGs relative to resting lupus neutrophils except for ARHGEF12 which is relative to resting healthy control neutrophils.

^b^ http://www.biir.net/public_wikis/module_annotation/V2_Trial_8_Modules_M5.15.

Given the high probability for false discovery of targets in this study, a second validation study was performed to verify our findings. Expression of 15 genes with the highest fold-change in ALN as compared to ANLN was assessed using NanoString technology. In addition, 11 IFN-induced genes were assayed to further investigate the association between the IFN-signature and LN. Comparison of all 170 SLE patients with the 22 healthy control samples revealed that 23 of these genes displayed significantly elevated mRNA levels in SLE patients, with all of the neutrophil-related and 11 of the 12 IFN-induced genes assayed demonstrating elevated expression (Fig 1B). Similar findings were observed when each of the three SLE patient subsets (for demographics and clinical characteristics see Table 1) were compared with healthy controls, except that the fold increase for neutrophil-related genes in patients with active LN was higher than that seen for the other two disease subsets. When active SLE patients with and without LN were compared, all but 1 of the genes differentially expressed by microarray that were assayed in the NanoString cohort were replicated. In contrast, there was no difference in IFN-induced gene expression between active SLE patients with and without LN. Differences in gene expression between active and remission LN patients were similar to those between active patients with and without LN, raising that possibility that the gene expression changes in LN patients correlate with active inflammation in the kidney.

Comparison of the expression levels of those genes with significantly increased mRNA abundance in active LN revealed that the levels of all of the genes previously identified to be enriched in LDGs except *ARG1* correlated strongly with each other (ρ between 0.8 and 0.9).

Data for representative genes are shown in Fig 1C. In general, the genes reported to be enriched in LDGs were elevated in all subsets of lupus patients as compared to controls, but were seen at considerably higher levels in patients with active LN. In contrast, *DAAM2*, an actin-binding protein involved in cell adhesion and cytoskeletal rearrangement that is highly expressed in neutrophils [26, 27], was only found at elevated levels in SLE patients with active LN. Essentially identical results were observed for those patients examined in both the microarray discovery and NanoString validation phases of the study.

In a secondary analysis of the NanoString data, we explored the association of several clinical and laboratory features with transcript abundance (Fig 1D). Consistent with previous work, there was an inverse association between the abundance levels of IFN-induced genes and patient age as well as serum complement, and a positive association with the SLEDAI-2K and dsDNA Ab levels [17, 28–30]. Abundance levels of a number of neutrophil associated genes also correlated positively with the SLEDAI-2K, possibly because a significant component of the SLEDAI-2K is derived from renal related descriptors. This is supported by the positive association between the mRNA abundance of most of the neutrophil associated genes and proteinuria, and, to a lesser extent, hematuria. However, there was no association with biopsy class, subclass, activity score or chronicity score in the subset of active LN patients who had paired renal biopsies. While treatment with anti-malarials or immunosuppressive drugs did not appear to have an impact on neutrophil-related gene expression, there was a positive association between GCS dose and the expression levels of these genes. Not unexpectedly, there was also a positive association between the neutrophil count and proportion of neutrophils within the white blood cell count and neutrophil-related gene expression in the SLE patients.

### The etiology of the neutrophil signature in ALN is multifactorial

Given that the transcript levels of the cluster of genes that overlapped with those previously reported to be associated with LN in pediatric lupus and enriched in the LDG subset were tightly correlated with each other, we generated a composite neutrophil score by summing the expression levels of these nine genes before further examining the association of this signature with additional clinical and laboratory variables. This approach is similar to that used successfully to examine clinical associations with the interferon signature in multiple studies and was favored over calculating a score based upon the proportion of significantly up-regulated genes, as has been used previously [8, 9] due to the smaller number of genes examined in our study (9 as compared to 22). There was a very strong correlation between our calculated neutrophil score and the percentage of overexpressed genes (ρ = 0.963).

**Fig 2:**
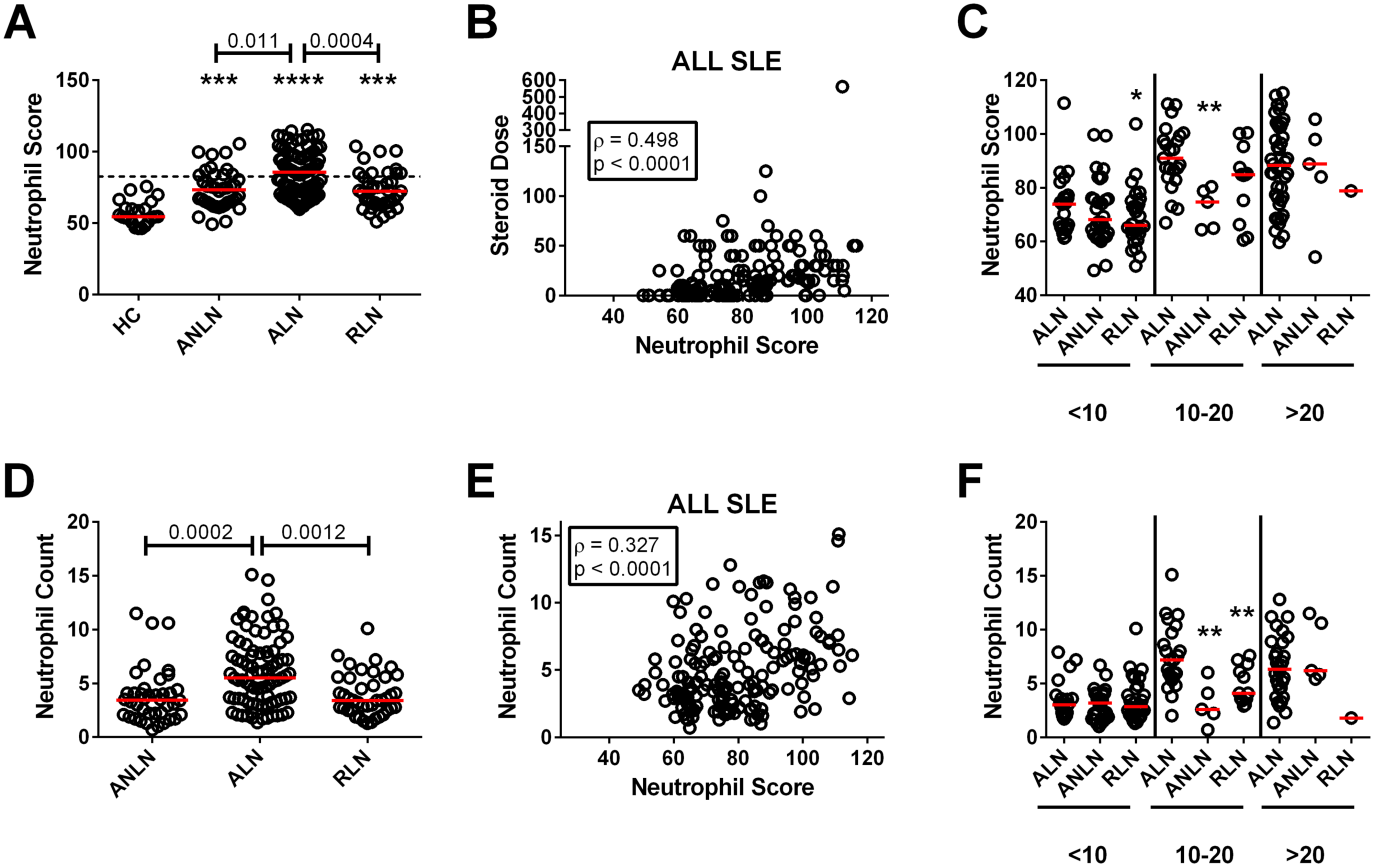
Association between the neutrophil score, GCS dose, and neutrophil count in the SLE patients. **(A)** Neutrophil scores in healthy controls (HC) and SLE patients stratified by disease group; active lupus without (ANLN) and with (ALN) nephritis, as well as lupus nephritis in remission (RLN). The dashed line represents 3 SD above the mean for HC. Significant differences from healthy controls are indicated by asterisks (*** p < 0.001, **** p < 0.0001), with the p values for significant differences between groups shown above the bars. **(B)** Association between GCS dose and the neutrophil score for all SLE patients. **(C)** Neutrophil scores stratified based upon GCS dose (shown at the bottom of the figure) with 3 groups <10 mg, 10–20 mg, and >20 mg (all GCS doses have been converted to their prednisone equivalent). Significant differences between the levels in ALN patients and other patient groups are shown (* p < 0.05, ** p < 0.01). **(D)** Neutrophil counts in SLE patients stratified by disease group. Significant differences between groups (p values) are shown above the bars. **(E)** Association between neutrophil count and the neutrophil score for all SLE patients. **(F)** Neutrophil counts stratified based upon GCS dose (shown at the bottom of the figure) with 3 groups <10 mg, 10–20 mg, and >20 mg (all GCS doses have been converted to their prednisone equivalent). Significant differences between the levels in ALN patients and other patient groups are shown (** p < 0.01).

As shown in Fig 2A, all three SLE patient subsets examined had elevated neutrophil scores as compared to healthy controls, with significantly elevated levels in ALN patients as compared to ANLN and RLN patients. Overall, 58.1% of ALN patients had an elevated neutrophil score (> 3 SD above the mean for healthy controls), which was a significantly increased proportion as compared to ANLN (27.5%, p = 0.0020) and RLN (24.4%, p = 0.0005) patients. As was seen for the individual genes, there was a significant correlation between the SLEDAI-2K and the neutrophil score. When the individual descriptors of the SLEDAI-2K were examined, the only significant associations were with hematuria (ρ = 0.187, p = 0.015), proteinuria (ρ = 0.328, p < 0.0001), pyuria (ρ = 0.203 p = 0.0084, and anti-dsDNA antibodies (ρ = 0.202, p = 0.0089), suggesting that the association with the SLEDAI-2K was predominantly driven by renal disease. For the 61 ALN patients that had a biopsy within 2 months of their sampling, there was no association between the neutrophil score and renal biopsy class, activity score, chronicity score, or the presence or absence of active proliferative lesions. Within the patients with ANLN, 8 had a prior history of LN and 3 subsequently developed LN on longitudinal follow-up. There was no association between an elevated neutrophil score and LN ever, prior LN, or subsequent development of LN (all p > 0.05, Fisher’s exact test), although there was a trend to an increased proportion of patients with high neutrophil scores developing a subsequent renal flare (18.2% as compared to 3.4% in patients with a low score). A similar trend was seen in the RLN patients (50% in patients with high score as compared to 34.8% with a low score), however this again did not achieve statistical significance.

Although, as previously observed for the individual genes, there was no association between anti-malarial or immunosuppressive treatment and the neutrophil score, a moderate correlation was seen with GCS dose (Fig 2B) and this remained present when each patient group was analyzed independently (ALN, ANLN, and RLN: ρ = 0.301, 0.286 and 0.425, respectively). To determine whether the neutrophil score in patients with ALN was significantly higher than patients with ANLN or RLN, who were on comparable doses of prednisone, patients were stratified into 3 groups (<10 mg, 10–20 mg, >20 mg). At lower doses of prednisone, ALN patients retained a trend to higher neutrophil scores that was marginally significant (Fig 2C), suggesting that both the ALN disease state itself and the higher doses of GCS treatment used to treat ALN contribute to the elevations of neutrophil scores observed. Notably, the neutrophil score remained elevated as compared to healthy controls in the 37 SLE patients that were off GCS at the time of disease flare (p < 0.0001) and this was seen both for patients with and without LN.

ALN patients had significant increases in their neutrophil counts as compared to ANLN and RLN patients (Fig 2D). Administration of GCS increases the neutrophil count and consistent with this there was a moderate correlation between GCS dose and the neutrophil count in all SLE patients (Fig 2E), which was also seen in each of the patient groups (ALN, ANLN, and RLN: ρ = 0.313, 0.415 and 0.364, respectively). When the groups were stratified for GCS dose, with the exception of patients on 10–20 mg of prednisone, differences between groups were no longer seen, suggesting that much of the difference in neutrophil counts between groups was due to GCS dose. In further support of this concept, a positive association between neutrophil count and neutrophil score was only seen from ALN patients (ρ = 0.342 as compared to ρ = 0.060 for ANLN and ρ = 0.028 for RLN patients). Indeed, in SLE patients off GCS the neutrophil score was inversely correlated with neutrophil count (ρ = -0.389, p = 0.017). Taken together, the data suggest that GCS dose and disease state predominantly contribute to the elevated neutrophil scores in patients with ALN as compared to ANLN and RLN, and in SLE patients as compared to healthy controls.

### Lack of correlation between the neutrophil signature and LDGs or neutrophil activation

As outlined previously, the genes comprising the neutrophil score are contained within the subset of genes that are enriched in LDGs [7, 24]. Given the modest association between the neutrophil count and the neutrophil score, we questioned whether the neutrophil score was better correlated with a specific property of the neutrophil population, such as the presence of LDGs or activated neutrophils, both of which have been proposed to lead to the elevated neutrophil signature in SLE [8, 24].

**Fig 3:**
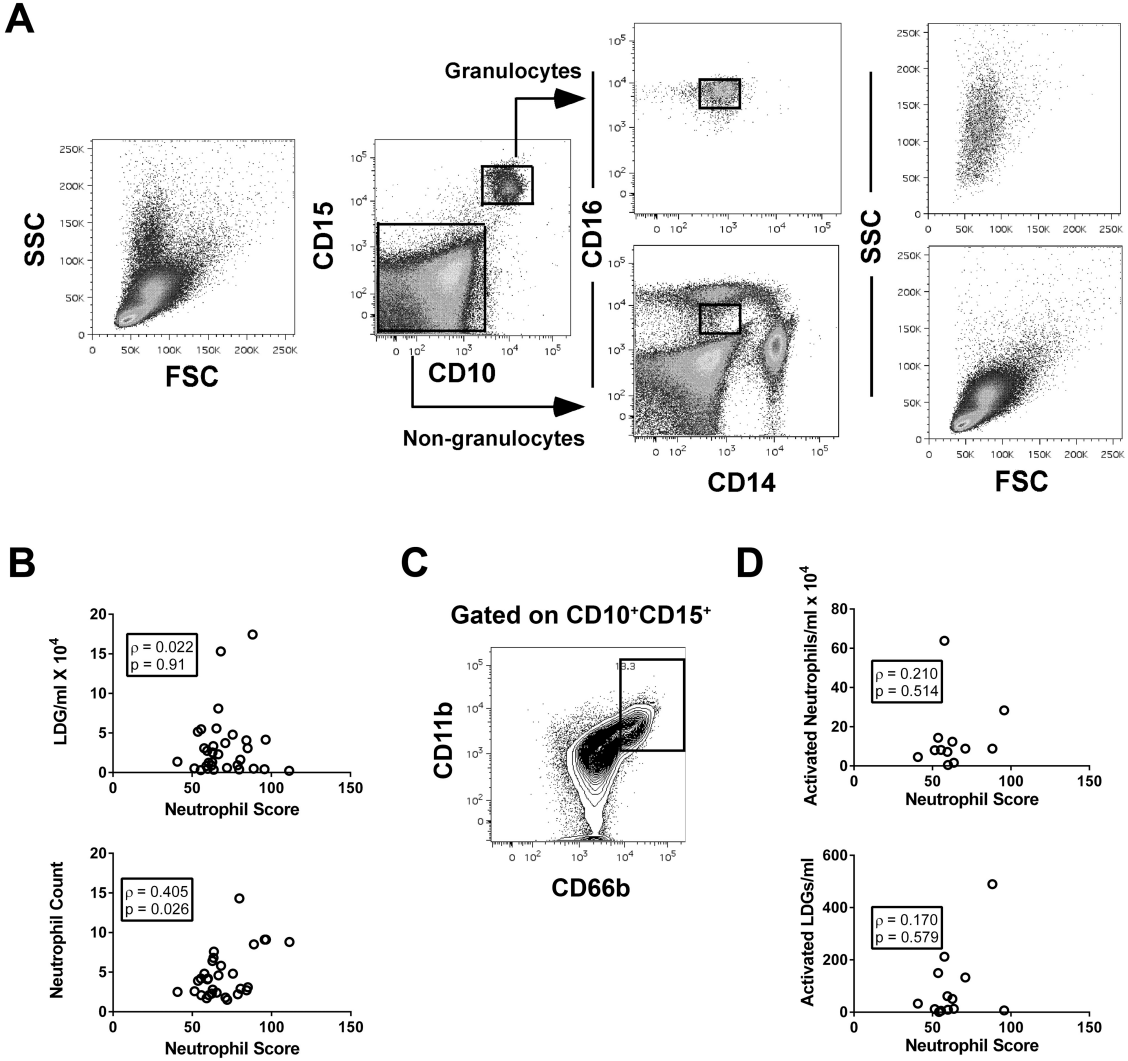
Correlation between the neutrophil score, LDGs, and neutrophil activation. **(A)** Flow plots showing the strategy for gating LDGs. PBMCs were isolated over a Ficoll gradient and then the CD10^+^CD15^+^ granulocytes gated as shown. As shown in the panels on the right, these cells were CD14^lo^CD16^+^ and had a unique forward (FSC) and side (SSC) scatter profile, consistent with the reported LDG phenotype [24]. **(B)** Correlation between the neutrophil score and the number of LDGs per ml or neutrophil count. **(C)** Flow plot showing the region used to gate activated (CD11b^hi^CD66b^hi^) cells within the whole peripheral blood CD10^+^CD15^+^ neutrophil population. **(D)** Correlation between the neutrophil score, the number of activated neutrophils or LDGs in the peripheral blood.

To address this question, PBMCs for 32 additional SLE patients were isolated over a Ficoll gradient and the LDGs identified by flow cytometry, as CD10^+^CD15^+^ cells (Fig 3A). There was no association between the neutrophil score and the number of LDGs per ml of blood (Fig 3B). In contrast, the same significant association between neutrophil score and neutrophil count that was observed in our original cohort was seen for this patient subset (Fig 3B). To determine whether there was an association between neutrophil activation and the neutrophil score, activated neutrophils were gated as CD11b^hi^CD66b^hi^ (Fig 3C). Although there was a weak non-significant association between the number of activated neutrophils in the whole peripheral blood or number of activated LDGs with the neutrophil score (Fig 3D) this was not as strong as the association with the neutrophil count for the same subset of patients (ρ = 0.634, p = 0.030). Furthermore, there was no correlation between the neutrophil score and the proportion of activated neutrophils within the blood or the LDG subpopulation (r = -0.140 or 0.129, respectively). Taken together, the data suggest that the elevated levels of neutrophil gene expression in lupus do not arise solely from the presence of LDGs or activated neutrophils in the peripheral blood.

### Longitudinal analysis of RNA abundance in lupus nephritis

**Fig 4:**
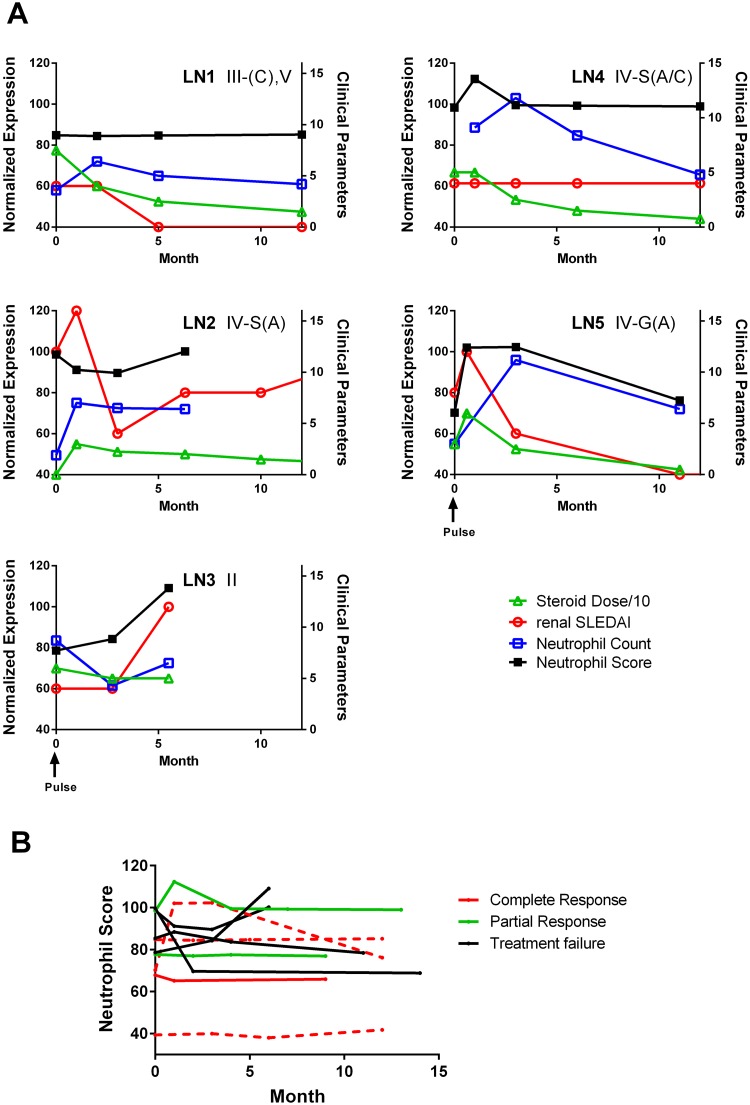
Correlation between neutrophil and clinical parameters over time. **(A)** Representative results showing the neutrophil score during longitudinal follow-up for five independent patients with LN. Scales on the left indicate neutrophil score, whereas those on the right give the values for the clinical parameters examined. The GCS doses were divided by 10 in order to enable them to be expressed on the same scale as the SLEDAI-2K and neutrophil counts. Numbers in the top right corner indicate the ISN renal biopsy class. **(B)** Comparison of changes in the neutrophil score over time in patients stratified by clinical response at 2 years following initiation of treatment for biopsy proven LN.

To further explore the association between the neutrophil score and various clinical parameters in patients with LN, we examined RNA abundance in a small number of lupus patients (n = 10) that had 3–4 serial determinations over an average of 10 months (range 6–15 months). Representative results for 5 patients are shown in Fig 4A. For over half of the patients (6/10), the neutrophil score remained relatively stable over the follow-up period (exemplified by LN1 and LN4). Notably, this stability occurred despite changes in prednisone dose and significant fluctuations in the neutrophil count. In the remaining patients, transcript abundance appeared to fluctuate with disease activity, either increasing with flares (see LN2 or LN3) or decreasing with treatment (see LN5). In one patient, the levels of neutrophil associated gene expression normalized with treatment despite the failure to achieve a clinical remission (Fig 4B). Overall, there were no consistent differences in the change in the neutrophil score over time between patients who achieved a partial or complete remission at 2 years and those that were treatment failures (Fig 4B).

## Discussion

In this study we found that the majority of genes that are expressed at significantly higher levels in active LN patients as compared to those without active LN are neutrophil-related genes. Although the presence of a neutrophil-derived signature in SLE was initially thought to be due to aberrant localization of a subset of neutrophils (LDGs) with PBMCs on a Ficoll gradient [7], more recently this signature has also been observed in the whole peripheral blood of both pediatric and adult SLE patients [8, 9]. We confirm this finding here. Indeed, the majority of genes that were elevated in active LN, as compared to active non-LN patients in our study, overlapped with those previously identified as part of the neutrophil signature in these two previous studies, and similarly to what was observed in these studies, we show that this signature is particularly elevated in patients with LN. Taken together, these findings indicate that the neutrophil signature in SLE is robust and reproducible. However, there are some important differences between our findings and those reported previously, particularly as pertains to the role of potential confounders, such as GCS therapy, and their impact on clinical associations. While elevated levels of neutrophil-related gene expression were seen in GCS naïve SLE patients, indicating that this signature is associated with the lupus disease state, we found a moderate correlation between GCS dose and the levels of neutrophil-related RNA abundance. Indeed, much of the difference observed between active LN and active non-LN was lost when the data was adjusted for GCS dose, suggesting that GCS dose is a major confounder in treated patients. As a trend to increased neutrophil scores remained in active LN as compared to remission LN and active non-LN patients at lower GCS doses, it is likely that there is also an independent association between the presence of active LN and an elevated neutrophil signature. However, further studies of untreated active adult SLE patients with and without LN are needed to definitively conclude that this is the case.

Although patients with active proliferative nephritis were previously reported to have higher neutrophil-derived RNA abundance as compared to those with non-proliferative lesions [9], the neutrophil score was not different between these two patient subsets in our study. This lack of difference was not due to the size or composition of our cohort, as the number of patients with paired biopsies in our study was 2.5 fold higher than that in the previous study (61 vs 24), with roughly half of our patients having active proliferative nephritis (n = 36), and the remainder having pure membranous (pure Class V, n = 11) or chronic/inactive lesions (n = 15). Therefore, we should have had substantially increased power as compared to the previous study to detect differences if they were present. However, there were some differences between the two studies with regard to the ethnicity of the patients and timing of the biopsies with respect to the blood draw. There was no difference in the neutrophil score between Caucasian and non-Caucasian subjects, or between proliferative and non-proliferative LN, when just the subset of Caucasians was examined. When we stratified our analysis based upon the timing of the blood draw relative to the biopsy, again the neutrophil score did not differ between proliferative and non-proliferative LN for patients who had their biopsy the same day as their blood draw, within 3 days of blood draw, or within 2 weeks after the blood draw. Nevertheless, because the blood draws were timed to the biopsy and not when the renal flare was first detected, many patients had already received treatment prior to the blood draw, which could have indirectly affected the results. To address this question, we compared active LN patients who were on < 20 mg of prednisone. This analysis revealed a marginally significant increase (p = 0.054) in the neutrophil score in patients with Class III/IV as compared to Class V changes on renal biopsy. Thus, it is possible that differences in the neutrophil signature between biopsy classes were obscured by treatment effects.

Not unexpectedly, a modest association between neutrophil-related gene expression (both score and individual genes) and neutrophil count was seen. This appeared to be largely driven by GCS dose, as it was not seen in patients off GCS nor was it observed in all patient subsets. These findings suggest that both the SLE disease state and GCS treatment promote neutrophil-related gene expression in SLE independently of the neutrophil count.

How do GCS increase neutrophil-related gene expression? GCS have been reported to elevate neutrophil counts by increasing release of cells from the bone marrow, demarginating neutrophils from vessel walls, and reducing apoptosis [31–33]. The first two of these processes have been shown to result in an increased proportion of immature cells within the neutrophil population in the circulation. Many of the genes that are enriched in LDGs are also expressed at high levels in immature neutrophils [34, 35]. Therefore, the association between GCS dose and neutrophil-related gene expression may result not only from the ability of GCS to increase the neutrophil count but also their ability to increase the proportion of immature cells within it. Notably, LDGs may also represent an activated immature neutrophil population, as a subpopulation of LDGs has been noted to be less segmented and more lobular than mature neutrophils [24, 25].

In addition to GCS, several other factors present in SLE could lead to an increased proportion of immature neutrophils within the peripheral blood. We and others have shown that a subset of SLE patients have elevated levels of serum GM-CSF [36, 37], particularly those with active disease, which could also increase the proportion of immature neutrophils leaving the bone marrow in patients with active SLE. In addition, factors that lead to increased destruction or consumption of neutrophils, or alternatively, impaired bone marrow production of neutrophils, could lead to a relative increase in the serum levels of G-CSF and GM-CSF [38], resulting in increased proportions of immature neutrophils in the peripheral blood through homeostatic mechanisms. In this context, it is possible that the association between lupus, and particularly active LN, and the neutrophil score results from increased destruction of neutrophils as a result of ongoing NETosis. Previous studies have shown that the neutrophils of SLE patients are more susceptible to NETosis through a sterile mechanism involving type I interferons and nuclear antigen containing immune complexes [39, 40], and there is increasing evidence that NETosis plays an important role in the pathogenesis of LN [25, 41].

Comparison of the neutrophil-related gene signature in active and remission LN showed reductions in the levels of these genes in patients in remission, suggesting that they might act as biomarkers for active LN. However, the neutrophil score remained stable in the majority of the patients with repeated measures over the first year following treatment, and fluctuations in the neutrophil score appeared to mirror renal disease activity in only a subset of patients. This finding, taken together with the overlap in neutrophil scores between patients with ALN and ANLN or RLN, as well as the potential confounding effect of GCS, suggests that the neutrophil score and other measures of neutrophil-related gene expression alone may have limited utility as biomarkers for active renal disease. Whether the levels of neutrophil-related gene expression provide additional information when considered in tandem with more conventional biomarkers, such as anti-dsDNA and complement, or urinary biomarkers, such as proteinuria and various pro-inflammatory cytokines, will require further study.

## Supporting information

S1 Table Genes differentially expression between active SLE patients and healthy controls. (PDF)
