# Supplementary material for: Identification of a neutrophil-related gene expression signature that is enriched in adult systemic lupus erythematosus patients with active nephritis: Clinical/pathologic associations and etiologic mechanisms
Source: PLoS One. 2018 May 9;13(5):e0196117. doi: 10.1371/journal.pone.0196117 (PMC5942792; doi:10.1371/journal.pone.0196117)
Supplement: S1 Table — (PDF) [file pone.0196117.s001.pdf]

S1 Table. Genes differentially expression between active SLE patients and healthy controls.

		Lupus vs Control		Renal vs Non-renal	
Gene Symbol	Gene Name	Fold-change (Log_2)	q value	Fold-change (Log_2)	q value
IFI27	interferon-alpha inducible protein 27	3.303274	0.023204	-0.72399	0.512609
IFI44L	IFN-induced protein 44-like	3.176316	0.023204	-0.9631	0.361021
NRIR	negative regulator of interferon response (non-protein encoding)	2.694824	0.023204	-0.7609	0.361918
RSAD2	radical S-adenosyl methionine domain containing 2	2.595622	0.023204	-0.69023	0.422425
IFI44	IFN-induced protein 44	2.550249	0.036486	-0.6765	0.445664
IFIT1	interferon induced protein with tetratricopeptide repeats 1	2.335846	0.083659	-0.68017	0.47248
CMPK2	cytokine monophospate (UMP-CMP) kinase 2, mitochondiral	2.215621	0.030238	-0.56137	0.456005
OAS3	2'-5' oligoadenylate synthease 3	2.186463	0.044115	-0.76725	0.347388
HERC5	HECT and RLD domain containing E3 ubiquitin protein ligase 5	2.081185	0.040468	-0.51354	0.500747
OAS1	2'-5' oligoadenylate synthease 1	1.975429	0.031879	-0.72232	0.296238
LY6E	lymphocyte antigen 6 complex , locus E	1.878187	0.060042	-1.01733	0.212435
CCL2	monocyte chemotatic proteins -1	1.844947	0.023204	-1.30351	0.154865
EPSTI1	epithelial stromal interaction 1	1.818155	0.042211	-0.30873	0.664127
IFI6	interferon-alpha inducible protein 6	1.768846	0.044115	-0.68225	0.306679
DDX60	DEXD/H box RNA helicase 60	1.761375	0.040468	-0.47369	0.459158
IFIT3	interferon-induced protein with tetratricopeptide repeats 3	1.718507	0.051104	-0.49202	0.448678
MX1	myxovirus resistance 1	1.67446	0.040468	-0.56973	0.349106
OAS2	2'-5' oligoadenylate synthease 2	1.65719	0.083659	-0.61326	0.361107
HIST1H3G	histone cluster 1, H3g	1.655218	0.040468	0.092961	0.894518
SIGLEC1	sialic acid binding Ig-like lectin 1, sialoadhesin	1.607836	0.051973	-0.52245	0.394001
XAF1	XIAP associated factor 1	1.500866	0.040468	-0.61302	0.274892
RNASE2	Rnase A Family, 2 (liver, eosinophil-derived neurotoxin)	1.477618	0.040468	-0.07797	0.902713
HERC6	HECT and RLD domain containing E3 ubiquitin protein ligase 6	1.461951	0.044115	-0.62684	0.265837
EIF2AK2	double-stranded RNA-dependent protein kinase	1.423925	0.023204	-0.35567	0.445455
USP18	ubiquitin specific peptidase 18	1.348654	0.023204	-0.48634	0.26856
RRM2	ribonucleotide reductase regulatory subunit M2	1.348315	0.051973	0.216724	0.693055
RTP4	receptor transporter protein 4	1.310735	0.040468	-0.48401	0.314328
PLSCR1	phospholipid scramblase 1	1.269274	0.040468	-0.34295	0.456493
SPATS2L	spermatogenesis associated serine-rich 2-like	1.227902	0.009411	-0.4488	0.230413
OASL	2'-5'-oligoadenylate synthetase like	1.182053	0.089906	-0.31785	0.513523
SAMD9L	sterile alpha motif domain containing 9-like	1.144724	0.023204	-0.31422	0.404008
LAP3	leucine aminopeptidase 3	1.116535	0.079059	-0.46154	0.306679
ISG15	ISG15 ubiquitin-like modifier	1.099692	0.094418	-0.34761	0.445664
